# Genetic Regulation of Physiological Reproductive Lifespan and Female Fertility

**DOI:** 10.3390/ijms22052556

**Published:** 2021-03-04

**Authors:** Isabelle M. McGrath, Sally Mortlock, Grant W. Montgomery

**Affiliations:** Institute for Molecular Bioscience, The University of Queensland, 306 Carmody Road, St Lucia, QLD 4072, Australia; isabelle.mcgrath@uq.edu.au (I.M.M.); s.mortlock@imb.uq.edu.au (S.M.)

**Keywords:** reproductive lifespan, fertility, genetic variation, FSH, AMH, menopause, review

## Abstract

There is substantial genetic variation for common traits associated with reproductive lifespan and for common diseases influencing female fertility. Progress in high-throughput sequencing and genome-wide association studies (GWAS) have transformed our understanding of common genetic risk factors for complex traits and diseases influencing reproductive lifespan and fertility. The data emerging from GWAS demonstrate the utility of genetics to explain epidemiological observations, revealing shared biological pathways linking puberty timing, fertility, reproductive ageing and health outcomes. The observations also identify unique genetic risk factors specific to different reproductive diseases impacting on female fertility. Sequencing in patients with primary ovarian insufficiency (POI) have identified mutations in a large number of genes while GWAS have revealed shared genetic risk factors for POI and ovarian ageing. Studies on age at menopause implicate DNA damage/repair genes with implications for follicle health and ageing. In addition to the discovery of individual genes and pathways, the increasingly powerful studies on common genetic risk factors help interpret the underlying relationships and direction of causation in the regulation of reproductive lifespan, fertility and related traits.

## 1. Introduction

Variation in reproductive lifespan and female fertility has implications for individual health, population size and ageing. Differences in reproductive lifespan, age-specific fertility rates, twinning frequency, and common diseases, such as polycystic ovarian syndrome (PCOS) and endometriosis, all contribute to reproductive outcomes [1,2,3]. Other factors influencing female fertility include the cumulative effects of environmental exposures and lifestyle.

Factors contributing to variation in reproductive traits and diseases include both genetic and environmental effects with genetic factors playing a major role in variation for the traits and diseases affecting reproductive outcomes. In the last decade, genome-wide association studies (GWAS) have mapped many common genetic risk factors responsible for this variation. Mapping studies are continuing, and the increasing GWAS sample sizes provide valuable data on genomic locations for genetic risk factors and the overlap in individual risk factors for related traits.

The purpose of this review is to outline, with examples, how these genetic studies are helping to understand the complex regulation of reproductive traits. It is not intended to provide a systematic review of all genetic risk factors influencing reproductive lifespan and female fertility. For more detailed coverage of genetic effects on individual traits and diseases, readers are referred to summaries in the GWAS catalog [4] and earlier reviews [2,3,5]. The review provides an overview of the genetic variation implicated in fertility-related hormone concentrations, reproductive traits and diseases and illustrates the complexity of links within and between reproductive traits together with methods to discover and analyse overlap between different traits and diseases and approaches to evaluate cause and effect for related conditions.

## 2. Genetic Effects on Reproductive Traits

Many reproductive traits show concordance between relatives; however, a simple Mendelian inheritance pattern is not present. Often these traits are complex, meaning they are influenced by many genetic variants of small effect (polygenic), in addition to an environmental component. A statistical method of assessing a genetic liability to a trait is by measuring heritability. Heritability is the proportion of phenotypic variance in the population explained by genetic variation, meaning that an estimate of 0.6 indicates that 60% of the variance in the trait within a population is due to genetic variation between individuals [5]. One of the most common methods of estimating heritability is by studying the differential discordance between identical (monozygotic) twins and non-identical (dizygotic) twins for the trait. Briefly, as both members of the twin pair are expected to have received similar environmental exposure during gestation and throughout childhood, a trait is likely to have a genetic component if monozygotic twins are more concordant for the trait than dizygotic twins. Other methods of estimation include pedigree-based studies. Further, the heritability estimates are dependent on the population—therefore estimates may vary across ethnic groups with differences in allele frequencies and exposures to different environmental factors. Many reproductive traits in women are known to have high heritability (Table 1).

Family-based studies estimate the magnitude of genetic variation, but do not identify specific genetic variants that contribute to the trait variation. GWAS provide a method to identify genomic regions containing the genetic variant/s influencing a trait. Increasing sample sizes has led to increased power, therefore enabling detection of regions with a small effect size. Studies investigating the variants contributing to age of menarche have greatly benefited from larger cohort sizes: in 2010, Elks et al. detected 32 genomic loci with a sample of 87,802 women [21]; in 2014, Perry et al. detected 106 genomic loci with a sample of 182,416 women [22]; in 2017, Day et al. detected 389 genomic loci with a large study of 368,888 women [23]. The trend to detecting larger numbers of risk loci with increasing sample size is also seen in other reproductive traits including endometriosis [24,25,26,27], and age at menopause [28,29,30,31].

Nevertheless, the regions identified thus far in GWAS account for only a small proportion of the variance predicted to arise from genetic factors. Another way to assess this is the single nucleotide polymorphism (SNP)-heritability which measures the proportion of phenotypic variance explained by a defined collection of SNPs. Day et al. (2017) estimated the SNP-heritability of age at menarche as 32% [23], while estimates of heritability, which accounts for more than the data for common SNPs, suggest 50–70% of variance in age at menarche is due to genetic risk factors. Therefore, many genetic variants contributing to variation in age at menarche have yet to be identified. This also applies to other reproductive traits. Estimates of SNP-heritability have been reported for age at first reproduction (0.15), age of menopause (0.06), endometriosis (0.26), uterine fibroids (0.33) and recurrent pregnancy loss (0.015) (Table 2), substantially lower than heritability estimates reported in Table 1 [31,32,33,34]. Although we are yet to fully understand the genetic contribution to these traits, it is clear genetics plays a significant role. With rapidly increasing sample sizes and advances in genomic technologies, we are becoming better equipped to understand the genetic complexity underlying reproductive traits.

## 3. Genetic Variation Regulating Reproductive Hormone Concentrations

**Figure 1:**
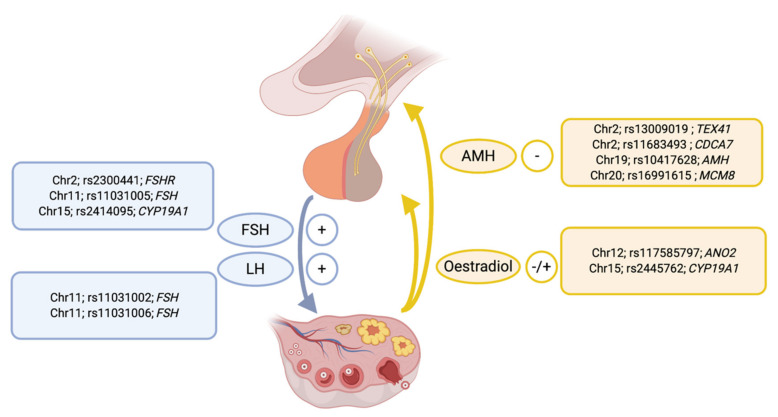
The location and lead SNP for genetic variants significantly associated with the measured concentrations of key hormones involved in hypothalamic-pituitary regulation of ovarian function. The nearest gene to the lead SNP for each genetic signal is also given.

Common genetic factors contributing to variation in concentrations of key hormones regulating reproductive function have been mapped through GWAS studies (Figure 1). The gonadotrophins follicle stimulating hormone (FSH) and luteinizing hormone (LH) play central roles within the hypothalamic-pituitary-gonadal axis. Common genetic risk factors are associated with variation in concentrations of both FSH and LH. Genome-wide significant association (*p* < 5 × 10^−8^) for both FSH and LH concentrations have been reported for three correlated variants (rs11031002, rs11031005, rs11031006) upstream of the gene (*FSHB*) encoding the β polypeptide for FSH [36,37]. Additional variants, rs2300441 on chromosome 2 located in an intron of the Follicle stimulating hormone receptor gene (*FSHR*) [38] and rs2414095 on chromosome 15 located in an intron of Cytochrome P450 Family 19 Subfamily A Member 1 (*CYP19A1*) [39] also influence FSH concentrations. The common variant rs2300441 explained considerably more variation in FSH concentrations than missense variants in *FSHR* reported previously [38].

GWAS studies have identified common variation influencing concentrations of both oestradiol and anti-Müllerian hormone (AMH) (Figure 1). Oestradiol concentrations are associated with different alleles for SNPs on chromosome 12 for rs117585797 in an intron of the Anoctamin 2 (*ANO2*) gene and on chromosome 15 for rs2445762 located in the third intron of *CYP19A1* [39]. Four variants were associated with variation in AMH concentrations (Figure 1). The strongest signal was a missense variant in the *AMH* gene (rs10417628) on chromosome 19 [40]. A variant for AMH concentrations at chromosome 20 (rs16991615) [41] near the Minichromosome Maintenance 8 Homologous Recombination Repair Factor (*MCM8*) gene also associated with natural age at menopause [28,29]. Other signals included variants on chromosome 2 near the Testis Expressed 41 (*TEX41*) gene and Cell Division Cycle Associated 7 (*CDCA7*) gene. The signal in AMH may be an artifact of the missense variant in *AMH* affecting the detection of the AMH protein in certain assays [40] but other variants are unlikely to be affected by this artifact as they are located on different chromosomes and not within the *AMH* gene.

## 4. Shared Genetic Risk Factors between Reproductive Traits and Diseases

The tight control of hormone concentrations is critical in the regulation of the female reproductive cycle. Therefore, it is not surprising that variants affecting hormone concentrations can also impact multiple reproductive traits. Here we highlight examples to illustrate the genetic overlap of related reproductive traits including specific examples where genetic variants in the same region influence multiple traits. In some cases, the same causal variant(s) influences multiple traits, while in other examples multiple signals near the same candidate gene appear to have independent effects on risk for individual traits suggesting complex temporal and tissue specific gene regulation in these regions. For a comprehensive list of variants implicated in fertility traits and reproductive diseases, readers are referred to the NHGRI-EBI GWAS Catalog [4].

### 4.1. FSHB Locus on Chromosome 11

FSH is synthesized and secreted by gonadotroph cells of the anterior pituitary gland and acts by binding to the FSH receptor (FSHR) [42]. The hormone is a heterodimer composed of the FSH-β chain together with an α-chain common to other members of the gonadotrophin hormone family [43]. In women, FSH plays an important role regulating antral follicle growth and recruitment of the dominant follicles(s) during each menstrual cycle that determine ovulation rate and twinning frequency [43,44].

**Figure 2:**
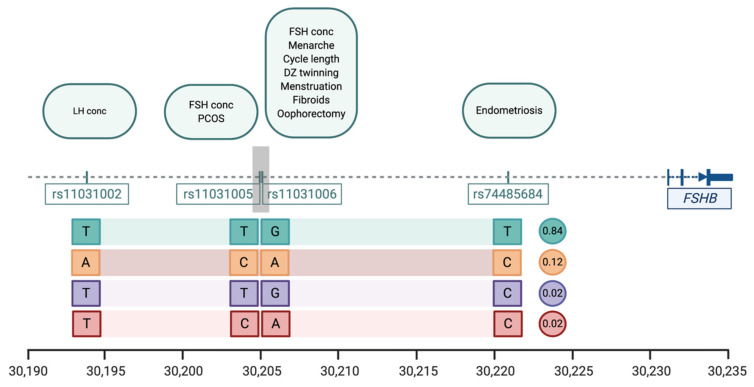
The location of SNPs significantly associated with reproductive traits and diseases from GWAS studies in the region immediately upstream of the *FSHB* locus on chromosome 11. The scale at the bottom of the figure is given in kilobases (kb) based on coordinates for human chromosome 11 build hg38. Lead SNPs identified in individual studies are shown below the dotted line and the individual traits and diseases associated with those lead SNPs are listed in the boxes above the line (see Table 3). The four SNPs are all highly correlated and haplotype analysis showing the association of alleles for individual SNPs identified four common allelic combinations or haplotypes with the expected frequencies of each haplotype shown in the circles to the right of each combination.

Genetic variation near *FSHB* is significantly associated with eleven traits and diseases including reproductive lifespan, menstrual cycle characteristics, FSH concentrations, endometriosis, polycystic ovarian syndrome, and uterine fibroids (Table 3). The genetic variants most strongly associated with individual traits and diseases show considerable overlap and consist of four SNPs located within a region of 37 kb region on chromosome 11p14 upstream of the promoter of *FSHB*, all highly correlated (Figure 2). The most common combination of alleles or haplotype (frequency 0.84, Figure 2) is associated with increased circulating FSH concentrations [37,44], increased frequency of dizygotic twinning [44], earlier age at menarche and menopause [23,31], shorter menstrual cycles, increased risk of endometriosis [27], and decreased risk of polycystic ovarian syndrome [45].

The lead SNP for DZ twinning rs11031005 is associated with increased FSH concentrations [37] and is strongly correlated with rs11031006 which is associated with several other reproductive traits and may have functional effects [21,24,28,32,45,46]. The SNPs rs11031005 and rs11031006 are also correlated with a promoter polymorphism (c.-211G > T, rs10835638; r^2^ = 0.67 with both SNPs) upstream of the transcription start site and reported to regulate *FSHB* gene transcription [47]. Women carrying the FSH decreasing GT genotype at rs10835638 had a more frequent poorer response to controlled ovarian hyperstimulation when compared to individuals with the GG genotype (47.4% vs. 26.5%, *p* = 0.010) [46]. The stronger association signals for several traits with rs11031002, rs11031005 and rs11031006 compared with the promoter polymorphisms suggests functional effects for one or more of these SNPs in regulating FSH concentrations.

The Combined Annotation Dependent Depletion (CADD) score [48] predicts functional or deleterious effects for SNPs. The CADD score for rs11031006 is 19.91, a high score for this index. This SNP (rs11031006) resides within a *FSHB* enhancer, 26 kb upstream of *FSHB*. This region exhibits open chromatin in the gonadotrope cells in the pituitary [49]. rs11031006 is able to upregulate *FSHB* expression in vitro: the minor (A) allele increases binding of Steroidogenic factor 1 (SF1) to the enhancer [49], a transcriptional activator of a number of genes in the hypothalamic-pituitary-gonadal axis [50]. Increased expression of *FSHB* with the minor allele was unexpected as this SNP has been associated with lower circulating FSH concentrations [51], however the in vitro conditions may affect the direction of response [49] and further studies are required to resolve these differences and determine if genetic variation at this enhancer is responsible for the effects on FSH concentrations and multiple reproductive traits and diseases.

The evidence is accumulating that common SNPs located in a functional element(s) upstream of the *FSHB* promoter regulate FSH concentrations with subsequent effects on multiple reproductive traits and diseases. However, effects acting through other genes in the region cannot be ruled out. SNPs correlated with the lead SNP increasing DZ twinning (rs11031005) extend across genes for both *FSHB* and ADP Ribosylation Factor Like GTPase 14 Effector Protein (*ARL14EP*). *ARL14EP* encodes an effector protein that interacts with ADP-ribosylation factor-like 14 (ARL14), beta-actin (ACTB) and actin-based motor protein myosin 1E (MYO1E) and controls the export of major histocompatibility class II molecules by connecting to the actin network [52]. It is expressed in a large number of tissues with relatively high levels in ovary, testis and the uterus. SNPs located in the transcription start site (TSS)/enhancer region of *ARL14EP* are highly correlated with rs11031005 and rs4071559 (LD with rs11031005, r^2^ = 0.82) is an eQTL for *ARL14EP* in the testis [53].

### 4.2. ESR1 Locus on Chromosome 6

The major biologically active oestrogen 17β-oestradiol has key roles in a multitude of organ systems in women. Oestrogen is involved in the development of secondary sex characteristics, in the regulation of the menstrual cycle (e.g., hormonal feedback and cell proliferation in the endometrium), and a decline in oestrogen levels is associated with menopause. Oestrogen is also involved in the muscular [55], central nervous [56] and skeletal systems [57]. The effects of oestrogen are mediated through its interaction with the oestrogen receptor (ER). There are two isoforms of ER: ERα is encoded by the *ESR1* gene (chromosome 6) [58], while ERβ is encoded by the *ESR2* gene (chromosome 14) [59]. Both show differential and overlapping expression across tissues and cell types [60]. Polymorphisms in the ER gene regions, particularly in the *ESR1* region, are implicated in risk for a variety of traits, such as breast cancer [61,62], age of menarche [22], age at first birth [63], uterine fibroids [54], endometriosis [27]. While many other variants in *ESR1* have been associated with other disorders by candidate gene studies [64,65,66,67,68,69], these findings have not been replicated by GWAS [20,22,70,71,72,73].

Analysis of correlations between variants in the *ESR1* region show SNPs associated with age at first birth, age at menarche or breast cancer are not likely to result from the same causal variants as the SNPs associated with endometriosis risk [74]. The lead uterine fibroid SNP (rs58415480) is strongly correlated with the endometriosis SNP (rs71575922) and risk for endometriosis and uterine fibroids at this locus may result from the same causal mechanism(s). Analysis of candidate gene studies for *ESR1* SNPs in endometriosis and comparison with the GWAS results show no evidence of independent association for the reported candidate gene SNPs in the GWAS results. The true signals in the region of *ESR1* associated with endometriosis risk are the non-coding variants located in intergenic regions flanking *ESR1* [27,74].

To evaluate the complex relationships between genetic variants and reproductive traits in the *ESR1* region, we analysed endometrial expression of genes from this region and correlated expression with changes in hormone concentrations and receptor expression changes across the menstrual cycle. We assessed patterns of expression for 15 genes within 2 Mb of the *ESR1* locus and identified a set of genes that show correlated changes in expression indicative of co-regulation with *ESR1*. The set included genes immediately upstream of *ESR1* (*RMND1*, *ARMT1*, *CCDC170*) and a gene (*FBXO5*) downstream of *ESR1* whose expression was significantly positively correlated with *ESR1* expression. The strongest evidence for correlated expression with *ESR1* was for *CCDC170* and together results suggest genes in the *ESR1* region may be co-regulated and not just menstrual cycle dependent. We found no evidence that the lead SNPs from the GWAS studies directly affect expression of any of these co-regulated genes. Results may be due to limited sample size or analysis of endometrial tissue with multiple cell types if the effects on gene expression are cell-type specific [74]. Further studies will be required to understand the complex nature of independent genetic signals in the *ESR1* region affecting multiple related reproductive traits. The results suggest complex regulation of gene expression in the *ESR1* region and genes other than *ESR1* should also be considered as potential target genes.

## 5. Age at Menopause

Another example of applications of GWAS data is understanding the complex relationships between age at natural menopause (ANM), ovarian reserve, declining fertility and AMH concentrations. Natural fertility decreases substantially some 10 years before menopause, partly related to a decline in reserve of primordial follicles in the ovary [2,31]. Earlier ANM is also associated with increased risk of osteoporosis [19,70,71], and increased risk of cardiovascular disease [75,76,77]. Later ANM is associated with increased risk of breast cancer [78], ovarian cancer [79,80], and endometrial cancer [81]. Genetic risk factors are known to contribute to ANM (Table 1). The largest GWAS performed for ANM involved 69,360 women, in which 54 independent signals from 44 genomic regions were identified. Pathway analysis indicated these regions show enrichment for DNA damage response genes and this may underlie the genetic links between ANM and breast cancer. Mendelian Randomisation analysis indicated the link between later ANM and increased breast cancer risk was a causal relationship (~6% yearly risk increase, *p* = 3 × 10^−14^) [31]. Mendelian Randomisation is a method that assesses a causal relationship between a risk factor (ANM) and an outcome (breast cancer) that is less likely to be affected by confounding factors than observational studies, by assessing the relationship between the genetic predisposition to the risk factor with the outcome [82].

Subsequent analysis of the overlap between the lead SNPs for ANM and genetic effects on gene expression in these regions identified 24 genes where there was overlap in signals and evidence the same causal variant may affect both expression of the gene and ANM [83]. These include eight genes in ANM associated regions previously annotated to DNA damage response pathways [31,83] suggesting the decline in the pool of available ovarian follicles contributing to declining fertility and the approaching menopause, may be related to reduced ability to repair DNA damage.

**Figure 3:**
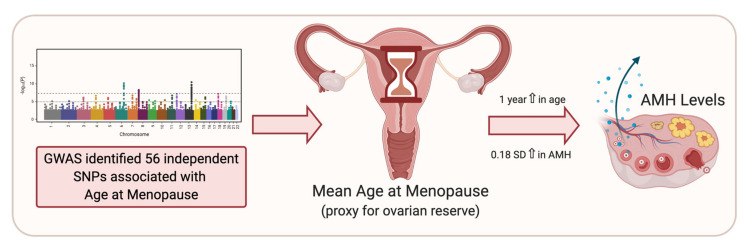
The application of Mendelian Randomisation to evaluate use of anti-Müllerian hormone (AMH) as a biomarker for ovarian reserve. Genome-wide association studies (GWAS) have identified 56 genetic variants associated with menopause timing [31]. Mendelian Randomisation was used to assess association of menopause timing (a proxy for ovarian reserve) with AMH concentrations [41] making use of the 56 genetic variants associated with age at natural menopause (ANM) to genetically predicted age at menopause. Earlier predicted ANM was associated with higher AMH concentrations supporting the use of AMH to measure ovarian reserve [41].

One reason for interest in regulation of the hormone AMH is because it is proposed as a marker of ovarian follicle reserve. In females, AMH is produced by the granulosa cells of growing follicles, meaning AMH levels reflect the number of growing follicles, and hence can be used to estimate ovarian reserve [84]. Recently, Ruth et al. (2019) investigated genetic effects on the expression of AMH in pre-menopausal women of late reproductive age [41]. As noted above, SNP rs16991615 in *MCM8* is associated with lower AMH concentrations and is a published variant associated with earlier menopause [41]. This SNP is a missense variant in exon 9 of *MCM8* (E341K) required for homologous recombination. The study utilised Mendelian Randomisation to assess association of menopause timing (a proxy for ovarian reserve) with AMH level. The genetically predicted age at menopause (estimated through the 56 genetic variants associated with menopause timing [31]) were associated with pre-menopausal AMH levels, suggesting AMH concentrations are predicting declining ovarian reserve in premenopausal women and genetic risk factors and/or AMH may help predict age at menopause (Figure 3) [41].

## 6. Summary and Conclusions

Genetic risk factors for common complex phenotypes like those discussed in this review are characterised by variants with small effects mostly located within introns and intergenic regions. This raises questions about how individual variants with small effects influence reproductive traits and fertility. It is thought that the causal variants are mostly located in genome sequences responsible for regulating epigenetic programming and gene expression, and influence disease risk through modifying this regulation. Numerous studies now document the genetic regulation of both gene expression [55,81,82] and methylation signals [85,86].

It is clear from the accumulated GWAS data that genetic risk for complex phenotypes is made up from the additive effects of 100 s or 1000 s of individual variants across the genome acting in an additive fashion [87]. Estimates of between one and two thirds of the heritability for common traits and diseases can be explained by the additive effects of common SNPs (SNPs with minor allele frequencies > 5%) [87]. There may be several reasons for the differences in estimates for heritability and SNP-heritability described earlier and shown more generally in other studies. Large-scale GWAS improves discovery of risk variants and provides better estimates for effect size for individual variants, thereby improving our estimates of SNP heritability. Some additive variation is due to causal variants with minor allele frequencies < 1%, which are typically not sampled in GWAS and could be substantial [87]. Effect sizes for the causal variants in each region may be underestimated because there may be more than one signal in a region, and we have yet to identify the true causal variant. In addition, effect sizes are mean effects across multiple studies that may have different criteria for disease diagnosis. Overall, genetic factors influence traits and diseases through the effects of many variants across the genome influencing common pathways [31].

Nevertheless, and despite small effects, specific variants like those reported for *FSHB* must have important effects because the same variant(s) are associated with many related traits. In other cases, like the region of *ESR1*, different independent variants influence different traits and diseases. We still have imperfect data about functional mechanisms to help understand how the same variant or region alters gene regulation sufficiently to affect many traits and diseases. For example, we have very limited data for genetic effects on gene expression or epigenetic regulation for critical tissues like the pituitary gland or ovaries [53]. There may be cell type specific effects or critical windows during development that alter later cell programming and we have even less data to help answer these questions. Effects of the critical variants on regulation of gene expression in specific cell types or at critical times may be greater than suggested from the estimate of genetic effects on disease outcomes from GWAS. Multiple independent signals in the *ESR1* region do suggest independent regulation of *ESR1* and/or other genes in the region in different tissues responsible for the different disease outcomes [74].

Reproductive traits and diseases are highly polygenic, as with most traits, and influenced by multiple genetic factors, some of which are shared between traits. GWAS datasets can be used to understand the complex regulation of reproductive traits through genetic correlation and Mendelian Randomisation studies. Examples discussed show genetic variation influences concentrations of key reproductive hormones which may in turn affect common variation in reproductive lifespan and risk for associated diseases. Genetic effects show that in some cases like the *FSHB* locus, the same causal variant(s) effect hormonal concentrations and multiple reproductive traits. In contrast at the *ESR1* locus, there appear to be multiple signals affecting reproductive traits independently suggesting complex regulation of *ESR1* and other genes in the immediate region in a tissue and possible time dependent fashion. Functional studies that identify the target genes and mechanisms to link established genetic variants to trait variation and disease risk are required to understand this complex regulation.

Genetic correlation and Mendelian Randomisation analyses help to understand overlap between related traits and diseases and the cause-and-effect relationships. Results show that the genetic factors regulating age at natural menopause are also associated with variation in ovarian reserve and AMH concentrations. These methods are being applied to understand the relationships between reproductive lifespan and fertility traits and effects of variation in reproductive lifespan on health. Increasingly powerful GWAS studies will provide greater precision, improving our capacity to detect and disentangle the complex web of variants controlling reproductive traits and diseases.

## Figures and Tables

**Table 1:** Estimates of heritability and 95% confidence intervals * for traits and diseases affecting reproductive lifespan and reproductive success.

Trait	Heritability Estimate (95% CI) *	Reference
Age at Menarche	0.49 (0.24–0.73)	[6]
	0.72	[7]
	0.57 (0.53–0.61)	[8]
	0.51 (0.46–0.55)	[9]
	0.72 (0.67–0.76)	[10]
Age at First Reproduction	0.24 (0.08–0.43)	[9]
Age at Menopause	0.45 (0.16–0.58)	[9]
	0.44 (0.36–0.50)	[11]
	0.63 (0.53–0.71)	[12]
	0.52 (0.35–0.69)	[13]
Hysterectomy	0.59 (0.43–0.72)	[12]
Endometriosis	0.47 (0.36–0.57)	[14]
	0.51 (0.36–0.66)	[15]
Uterine Fibroids	0.55 (0.46–0.63) ^&^	[16]
	0.69 (0.49–0.83) ^$^	[12]
PCOS	0.79	[17]
Pre-eclampsia	0.31 (0.09–0.45)	[18]
	0.54 (0.00–0.71)	[19]
Recurrent pregnancy loss	0.29 (0.20–0.38)	[20]

* 95% confidence intervals are given where these were provided in the original manuscripts; & Hospitalisations due to uterine fibroids; $ Fibroid status.

**Table 2:** Estimates of the SNP Heritability for reproductive traits from Table 1 where these are available.

Trait	SNP Heritability Estimate (Standard Error)	Reference
Age at menarche	0.32 (0.01)	[23]
Age at first reproduction	0.15 (0.04)	[32]
Age of menopause	0.06 (0.02)	[31]
Endometriosis	0.26 (0.04)	[33]
Uterine fibroids	0.33 (0.18)	[34]
Recurrent pregnancy loss	0.02 (0.40)	[35]

**Table 3:** Genetic association for common traits and diseases immediately upstream of the follicle stimulating hormone subunit beta (*FSHB*) on chromosome 11p14.1.

Trait	SNP	Position *	Pval	Effect Alleles ^&^	Study
Menstrual cycle length	rs11031006	30,204,981	1.1 × 10^−38^	A > G	[20]
Age at menarche	rs11031006	30,204,981	8.49 × 10^−14^	A > G	[23]
Age at menopause	rs11031006	30,204,981	8.5 × 10^−14^	A > G	[31]
Dizygotic twinning	rs11031006	30,204,981	1.25 × 10^−10^	G > A	[44]
FSH concentrations	rs11031005 rs11031006	30,204,809 30,204,981	1.74 × 10^−8^ 2.3 × 10^−10^	T > C G > A	[37] [44]
LH concentrations	rs11031002	30,193,714	3.94 × 10^−9^	T > A	[37]
Endometriosis	rs74485684	30,220,740	2.00 × 10^−8^	T > C	[27]
Polycystic ovarian syndrome	rs11031005	30,204,809	8.66 × 10^−13^	C > T	[45]
Excessive, frequent and irregular menstruation	rs11031006	30,204,981	1.1 × 10^−38^	A > G	[20]
Uterine fibroids	rs11031006	30,204,981	5.7 × 10^−15^	A > G	[54]
Bilateral oophorectomy	rs11031006	30,204,981	1.1 × 10^−38^	A > G	[20]

The LD between each listed SNP has an r^2^ > 0.82, except for rs11031002 and rs74485684, which have an r^2^ of 0.69 (European population); * Position on chromosome 11 (GRCh38.p12); & Direction of effect (allele increasing trait value or disease risk > alternative allele).

## Data Availability

LDlink (https://ldlink.nci.nih.gov/ accessed on 3 March 2021) database was used to determine the linkage disequilibrium (r^2^) between variants in Table 3.
